# Resting-state functional connectivity and quantitation of glutamate and GABA of the PCC/precuneus by magnetic resonance spectroscopy at 7T in healthy individuals

**DOI:** 10.1371/journal.pone.0244491

**Published:** 2020-12-29

**Authors:** Ofer M. Gonen, Bradford A. Moffat, Patrick Kwan, Terence J. O’Brien, Patricia M. Desmond, Elaine Lui

**Affiliations:** 1 Department of Neurology, The Royal Melbourne Hospital, Victoria, Australia; 2 Department of Medicine and Radiology, The University of Melbourne, Victoria, Australia; 3 Department of Neurology, The Alfred Hospital, Victoria, Australia; 4 Department of Neuroscience, Central Clinical School, Monash University, Victoria, Australia; 5 Department of Radiology, The Royal Melbourne Hospital, Victoria, Australia; University of Cambridge, UNITED KINGDOM

## Abstract

The default mode network (DMN) is the main large-scale network of the resting brain and the PCC/precuneus is a major hub of this network. Glutamate and GABA (γ-amino butyric acid) are the main excitatory and inhibitory neurotransmitters in the CNS, respectively. We studied glutamate and GABA concentrations in the PCC/precuneus via magnetic resonance spectroscopy (MRS) at 7T in relation to age and correlated them with functional connectivity between this region and other DMN nodes in ten healthy right-handed volunteers ranging in age between 23–68 years. Mean functional connectivity of the PCC/precuneus to the other DMN nodes and the glutamate/GABA ratio significantly correlated with age (*r* = 0.802, *p* = 0.005 and *r* = 0.793, *p* = 0.006, respectively) but not with each other. Glutamate and GABA alone did not significantly correlate with age nor with functional connectivity within the DMN. The glutamate/GABA ratio and functional connectivity of the PCC/precuneus are, therefore, independent age-related biomarkers of the DMN and may be combined in a multimodal pipeline to study DMN alterations in various disease states.

## Introduction

The Default-Mode Network (DMN) is the main large-scale network of the resting brain. It was first reported in 1997 after a series of PET studies demonstrated areas that consistently decrease their activity in various tasks in comparison with quiet repose (either with the eyes closed or with simple visual fixation) [1]. This has since been widely evaluated using resting state fMRI, which measures the functional connectivity between different brain regions belonging to the same network, as reflected by the coherent oscillations in their Blood Oxygenation Level Dependent (BOLD) contrast. Altered DMN function has been described in various disorders, such as Alzheimer’s disease, depression, schizophrenia, Attention Deficit Hyperactivity Disorder (ADHD), and epilepsy [2]. Its structural core is the Posterior Cingulate Cortex (PCC)/precuneus, and other main nodes are the right and left inferior parietal lobules of the lateral parietal cortices (mainly in the angular gyri) and the Mesial Prefrontal Cortex (MPFC) [3,4]. Other cortical and subcortical structures are also associated with the DMN [5]. For example, the PCC/precuneus is also known to be strongly connected, both structurally and functionally to the mesial temporal lobes, with a diurnal variation of functional connectivity [6,7].

Glutamate and GABA (γ-amino butyric acid) are the major excitatory and inhibitory neurotransmitters in the human brain, respectively [8,9]. Both metabolites can be quantified *in vivo* using MRS. While interest in quantitative magnetic resonance spectroscopy of these metabolites in the human brain is increasing, individual quantification of glutamate and GABA has been limited by overlapping resonances from other molecules at magnetic fields up to 4 Tesla [10]. MRS quantification of glutamate and GABA at the PCC/precuneus is of interest, as a main node of the resting-state default mode network of the brain and its implication in a wide range of neurological and psychiatric disease states [11,12].

Previous studies evaluating both resting state fMRI and MRS of the DMN have yielded conflicting results. Kapogiannis and coworkers studied the relationship between the intrinsic functional connectivity of the DMN at 3T, which was extracted via independent component analysis of the concentrations of glutamate and GABA in the PCC/precuneus in 20 healthy men. They used a midline 25*18*20 mm voxel which was placed with the intent to ensure maximum inclusion of the precuneus. Depending on individual anatomical variability, the voxel also included part of the PCC. Based on their findings, after controlling for age and grey matter volume in the MRS voxel, the concentrations of glutamate and GABA explained about half of the variance in DMN intrinsic functional connectivity [13].

Glutamate and GABA correlated positively and negatively with the functional connectivity, respectively. Positive correlation to the intrinsic functional connectivity was also discovered for the ratio of glutamate to GABA, which was thought to reflect the balance between excitation and inhibition in the region of interest. Interestingly, age did not independently affect DMN intrinsic functional connectivity [13].

In contrast, Arrubla et al, also at 3T, did not find any correlation between the intrinsic functional connectivity of the PCC/precuneus to the concentration of glutamate in a study on 31 healthy men using a 25*25*25 mm midline voxel [14]. A multimodal study of the DMN combining fMRI, MRS, EEG, and PET in 11 healthy male volunteers at 3T found a correlation between the mean BOLD signal in the DMN and the concentrations of creatine-scaled glutamate and GABA in the PCC and precuneus [15].

In a 7T study on 13 healthy volunteers, fMRI and occipital cortex MRS (20*20*20 mm midline voxel centered along the calcarine sulcus) were acquired during a short block of flickering checkerboards. While glutamate concentration correlated with the BOLD signal during visual activations, no such correlation was found in the resting state [16].

With better spectral resolution, the reliability of MRS, particularly of J-coupled metabolites including glutamate and GABA, has been shown to be superior at 7T compared to 3T [10]. Similarly, ultra-high field at 7T allows improved temporal and spatial resolution resting state fMRI imaging whilst achieving full brain coverage [17,18]. We therefore sought to determine in this study the correlation between the concentrations of glutamate and GABA in the PCC/precuneus and its functional connectivity with the main nodes of the DMN in healthy subjects at 7T.

An MRS study of normal aging and Alzheimer’s disease in humans at 3T, focusing on the anterior cingulate cortex and right hippocampus, demonstrated decline in GABA+ (GABA and macromolecules) as well as GLX+ (glutamate and glutamine) in these regions, thus underscoring the importance of elucidating exact changes of glutamate and GABA concentration in the PCC/precuneus, a task which can be achieved with 7T MRS [19].

Notably, a reduction in the ratio of glutamate/GABA in the brain of aging rodents has been previously described [20]. Given this finding, we hypothesized that a similar reduction in this ratio would be discovered in our study.

Most of the initial studies of resting state functional connectivity across the lifespan found reduced connectivity within the DMN in healthy elderly adults relative to young adults. Therefore, we also sought to examine the effect of age on the functional connectivity of the PCC/precuneus region as measured at 7T [21]. We hypothesized that functional connectivity would negatively correlate with age and that this would be at least partially reflected by the declining glutamate to GABA ratio.

## Methods

### Study subjects

Ten healthy, right-handed, volunteers were recruited from the staff of The Royal Melbourne Hospital and The University of Melbourne. The participant population consisted of 6 women and 4 men, with a mean age of 40.0±14.4 years (median 36.5). All participants had no history of neurological or psychiatric disease. The study was approved by the institutional review board of the University of Melbourne. All participants provided written informed consent.

### Imaging protocol

MRI scans were acquired using a 7T research scanner (Siemens Healthcare, Erlangen, Germany) with a 32-channel head-coil (Nova Medical Inc, Wilmington MA, USA). MP2RAGE (magnetization prepared 2 rapid gradient echoes), a three-dimensional T1-weighted sequence, was performed to assist with MRS voxel localization. The MP2RAGE before the first MRS acquisition was of 0.9 mm isotropic resolution, TR = 4900 ms, TE = 2.9 ms, TI1 = 700 ms, FA1 = 5°, TI2 = 2700 ms, FA2 = 6°. Resting state fMRI was then acquired using visual fixation (time of acquisition– 5:18 minutes).

All fMRI imaging was performed using a gradient echo multi-band EPI sequence, with multiband and parallel imaging acceleration [22]. A total image acceleration factor of 12 (2 by parallel and 6 by multi-band) and blipped-controlled aliasing was used to achieve whole brain coverage with 1.6 mm isotropic spatial resolution, and a temporal resolution of 800 ms with 300 repetitions (FOV = 208 mm, TE = 22.2 ms, FA = 45°).

**Fig 1:**
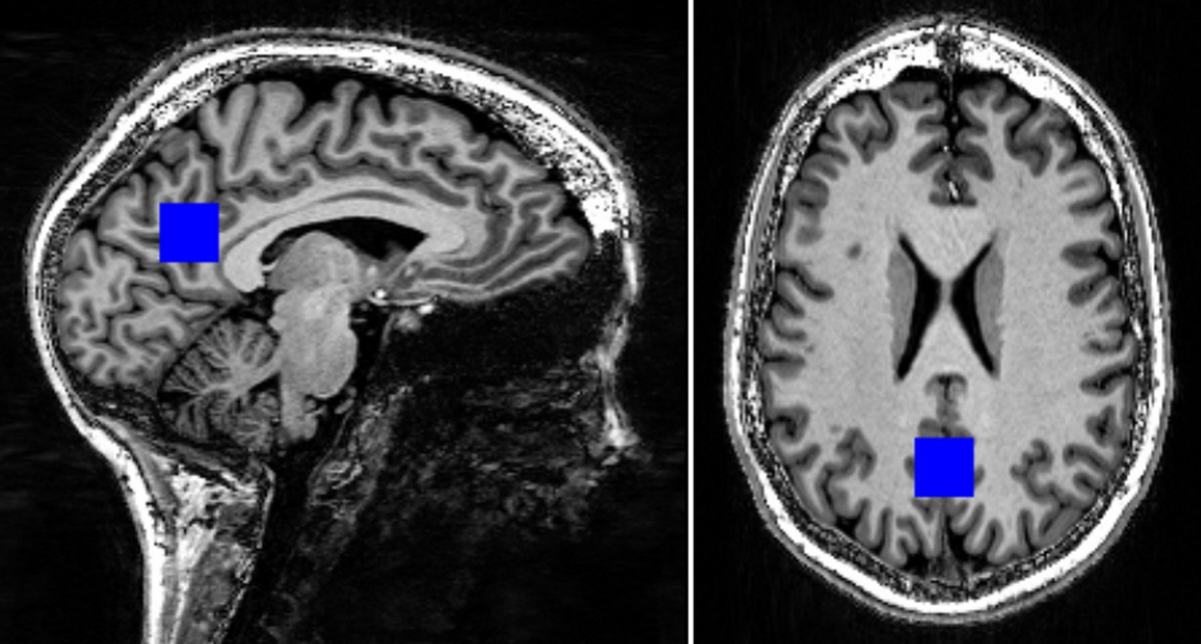
T1-weighted MRI with superimposed MRS 8 cm^3^ voxel. (left–parasagittal; right–axial).

After automated shimming followed by manual manipulation of the x, y, and z gradients to optimize line width, single voxel ^1^H MRS was acquired using the STEAM method with a 20*20*20 mm^3^ cubical voxel (volume of 8 mL). The voxel was located in the midline, with its anterior inferior border just above the corpus callosum, its anterior superior border just dorsal to the marginal branch of the cingulate sulcus, and its posterior border ventral to the parieto-occipital sulcus, in a similar manner to the study of Kantarci et al (Fig 1) [23]. The participants’ overlapping voxel localizations appear in the supplementary material–S1 Fig.

The following parameters were employed: TR = 8500 ms (except for participant 5, for which TR = 9300 ms was used due to reaching a SAR limit), TE = 6 ms, thirty-two averages for the water-suppressed sequence (acquisition time (TA) = 4:49 minutes) and four averages for the unsuppressed sequence (TA = 51 seconds). Although 2 MP2RAGE anatomy scans and 2 MRS were acquired for reproducibility evaluation, for the purpose of this study, only the first spectrum, acquired immediately following the fMRI, was used.

### Functional MRI pre-processing

Pre-processing of the fMRI data was performed using the SPM12 software (http://www.fil.ion.ucl.ac.uk/spm/software/spm12) and CONN toolbox version 19b on a platform of MATLAB Runtime 2019b – 9.7 (The MathWorks, Natick, MA, USA) [24].

The first ten functional datasets were excluded to reach steady-state magnetization and allow participants to adapt. All functional images were realigned to the mean volume using b-spline interpolation. Images were then unwarped via resampling of functional data along the phase-encoded direction to correct deformations caused by field inhomogeneities using the method described by Andersson et al and translated to center coordinates [25]. All images were normalized to the Montreal Neurological Institute (MNI) standard space using isotropic 2 mm resolution [26]. Motion parameters from realignment were evaluated and a motion artefact threshold (0.5 mm) was employed for exclusion of outliers. Spatial smoothing was performed with a 6 mm full width half maximum Gaussian kernel filter. The choice of 6 mm was based on using a size three times larger than the voxel resolution (2 mm) in order to optimize the signal-to-noise ratio [27].

The MP2RAGE anatomical images were used for co-registration, and were segmented into gray matter, white matter and cerebrospinal fluid implemented using SPM12. BOLD signal noise from the white matter and CSF was characterized with the principal component-based noise-correction ‘CompCor’ method from the CONN toolbox version 19b using 5 components for CSF and 15 components for white matter based on quality assurance plots to optimize physiological noise reduction [28]. Band-pass filtering was performed with a frequency window of 0.009 to 0.08 Hz.

**Fig 2:**
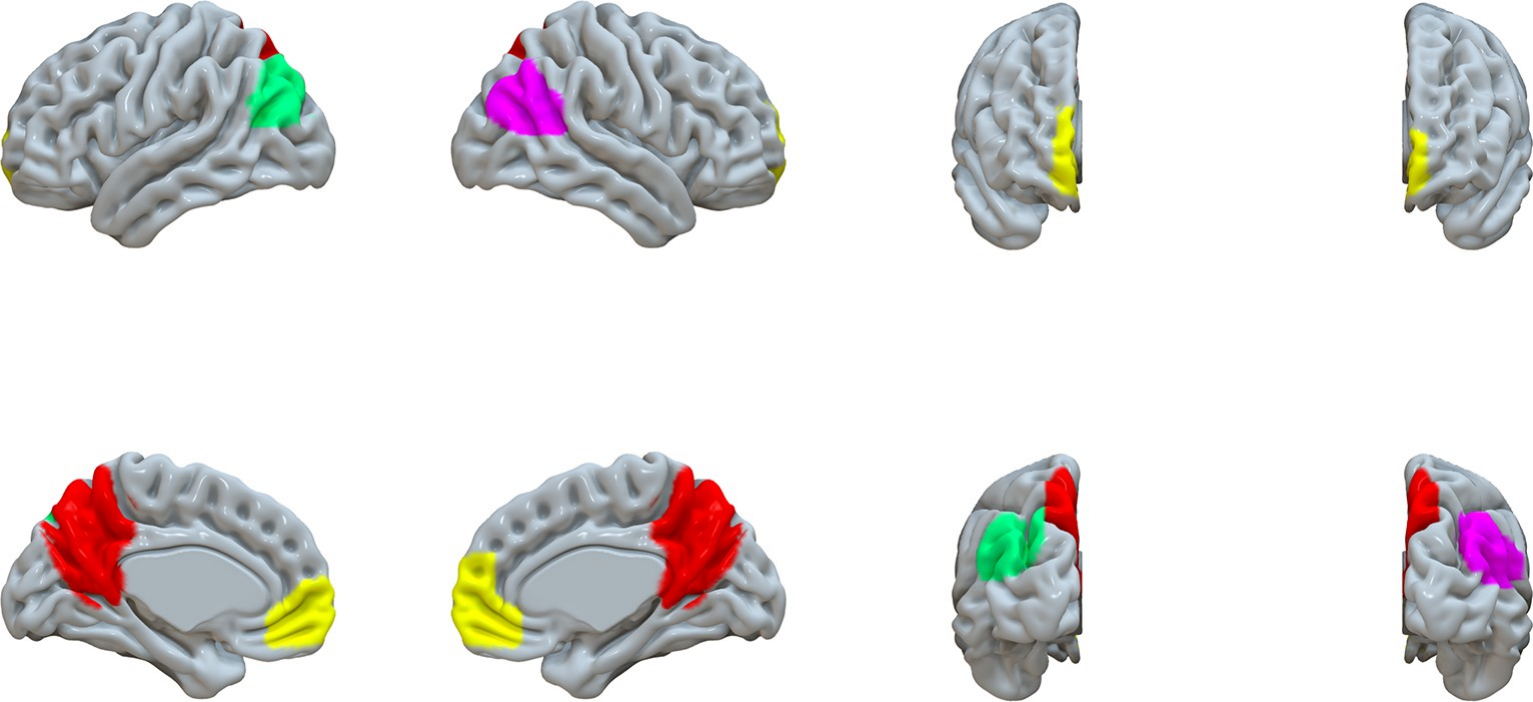
Regions of interest of cortical networks as used in the CONN. (Red–PCC/Pr; Yellow–MPFC; Green–left IPL; Magenta–right IPL).

Predefined Regions-Of-Interest (ROIs) were chosen based on extraction of the DMN via independent component analysis of the resting-state data of participants from the Human Connectome Project embedded in the CONN-fMRI Functional Connectivity toolbox (https://web.conn-toolbox.org/). The ROIs consisted of the following regions: PCC/precuneus, right inferior parietal lobule, left inferior parietal lobule, and medial prefrontal cortex (MPFC). MNI coordinates for these ROIs (x, y, z) were (1, -61, 38), (47, -67, 29), (-39, -77, 33), and (1, 55, -3), respectively–see Fig 2.

ROI-to-ROI functional connectivity maps were created for each subject. Maps of functional connectivity were calculated by computing Pearson’s product moment correlation between each pair of ROIs. For each ROI, the values were the mean of all voxels within the ROI. Correlation coefficients were converted to Z values using Fisher’s Transformation. For ROI-to-ROI analyses a threshold of *p* ≤ 0.001 was used for bidirectional explorations of connectivity (i.e. positive and negative associations). Results of exploratory analyses were considered significant if they survived correction for multiple comparisons (*p*-FDR ≤ 0.05).

In order to verify results obtained with the seed-based analysis outlined above using a data-driven approach, independent component analysis (ICA) using 20 components and a dimensionality reduction of 64 (default setting in CONN) was employed, based on the methods described by Calhoun et al [29]. Using the automatic correlational spatial match-to-template approach a component was extracted that maximally matched the DMN template stored in CONN (ICA5), with clusters passing the threshold of *p*-FDR<0.05 (one-way t-test for positive correlation) matching the PCC/precuneus and the right and left IPLs. A second component (ICA2), matching the DMN to a lesser extent but not matching any other resting-state network, was extracted, the largest cluster of which matched the MPFC. The ICA2 and ICA5 components are illustrated in the supplementary figures (S2 & S3 Figs). The chosen clusters from these components are described in the S1 Table. The functional connectivity between the PCC/precuneus, the right and left IPLs and the MPFC was averaged in a similar manner to the calculation performed for the seed-based data.

### Quantitative MRS calculation

Metabolite concentrations were quantified using LCModel version 6.3-0B with a 7-Tesla basis set [30]. Eddy-current correction was used, and the metabolite concentrations were scaled to unsuppressed water. Cramér–Rao lower bounds (CRLB), estimated standard deviations, expressed in percent of the estimated concentrations, were calculated by LCModel [30].

For partial volume effect correction, binary masks were created for each MRS voxel acquired using a MATLAB script created by Mr. Bartosz Kossowski from The Polish Academy of Sciences (https://www.nitrc.org/projects/rda2nifti/). MATLAB version R2017b was used (MathWorks, Natick, MA, United States). The dimensions of the masks were transformed via the ANTs toolbox (http://stnava.github.io/ANTs/) version 2.3.1 to be consistent with MP2RAGE coordinates [31]. The MP2RAGE anatomical images were skull-stripped and the brain extracted using FMRIB’s Brain Extraction Tool [32]. Each brain was then segmented using FMRIB’s Automated Segmentation Tool (FAST) into partial volume maps for gray matter, white matter and CSF [33].

The fractions of each of these partial volume maps within each MRS voxel were determined using the fslstats utility of the FSL suite version 5.0.10 [34]. The NMR-visible water concentration (mM) in the voxel was estimated by (43300f_GM_+35880f_WM_+55556f_CSF_)/(1-f_CSF_), where f_GM_, f_WM_, and f_CSF_ are the volume fractions of gray matter, white matter, and CSF in the voxel, and 43300, 35880, and 55556 are the millimolar concentrations of water in these tissues, respectively, based on the LCModel manual [35].

### Statistical analysis

Simple linear regression was employed to analyze the relationship between age and several dependent variables that were chosen based on prior studies: Functional connectivity of the PCC/precuneus, concentrations of glutamate and GABA, and the ratio of glutamate/GABA in the voxel of interest. In addition, the relationship between functional connectivity and the concentrations of glutamate, GABA, and the ratio of their concentrations was studied. In order to avoid type I error, a threshold of *p* = 0.05 was set for statistical significance.

All statistical analyses were conducted with Stata 15.0 (StataCorp, College Station, TX, USA).

## Results

**Fig 3:**
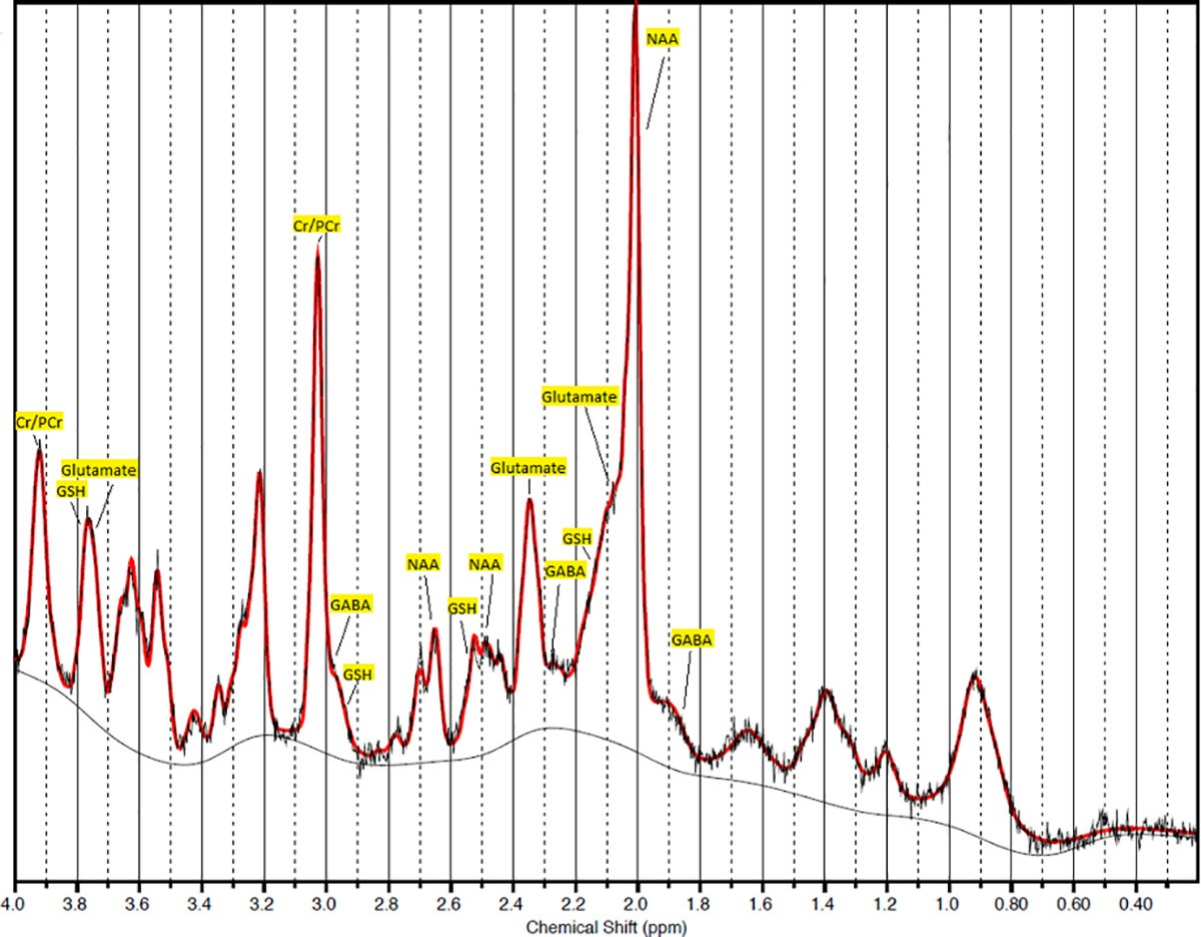
Example of LCModel output spectrum. (Cr = creatine; PCr = phosphocreatine; GSH = glutathione; GABA = γ-amino butyric acid; NAA = N-acetylaspartic acid).

Maximal CRLB for glutamate and GABA were 2% and 11%, respectively. Mean(±SD) values for these metabolites were 2%±0% and 8%±1.3%. The mean(±SD) Full Width at Half Maximum of the spectra was 0.03±0.003 ppm (8.9±0.9 Hz). Overall, these findings reflect good quality acquisition [36]. See Fig 3 for an example of an LCModel output spectrum.

Mean(±SD) values for the concentrations of glutamate and GABA were 10.3(±0.9) and 1.5(±0.2) mM, respectively. The mean ratio between glutamate and GABA was 7.0(±0.8). The mean Z value of the functional connectivity of the PCC/precuneus to the other 3 main DMN nodes in the seed-based approach was 0.7(±0.3). The ICA-based mean functional connectivity of the PCC/precuneus to the IPLs was 0.8(±0.3). The results for all 10 participants are shown in Table 1 and for all 11 linear regression in Table 2.

**Table 1:** fMRI and MRS results.

Participant	Age (years)	Glutamate (mM)	GABA (mM)	Glutamate/GABA	Mean Z Value (SB)	Mean Z Value (ICA)
1	27	10.5	1.7	6.2	0.5	0.5
2	39	10.8	1.7	6.4	0.6	0.7
3	68	9.1	1.1	8.3	1.5	1.4
4	36	10.4	1.4	7.4	0.7	0.8
5	53	10.7	1.3	8.2	0.6	0.7
6	23	9.8	1.5	6.5	0.7	0.7
7	56	12.5	1.7	7.4	1.1	0.9
8	31	10	1.5	6.7	0.6	0.8
9	37	10.1	1.7	5.9	0.7	1.2
10	30	9.5	1.4	6.8	0.3	0.4

(ICA = independent component analysis; SB = seed-based).

**Table 2:** Linear regressions.

No.	Dependent Variable	Independent Variable	β	95% CI Lower Limit	95% CI Upper Limit	r	*p*-value
1	Glutamate	Age	0.123	-0.039	0.064	0.192	0.596
2	GABA	Age	-0.007	-0.017	0.004	-0.472	0.168
3	Glutamate/GABA	Age	0.045	0.017	0.073	0.793	**0.006**
4	Mean Z Value (SB)	Age	0.019	0.007	0.030	0.802	**0.005**
5	Mean Z Value (SB)	Glutamate	0.017	-0.279	0.312	0.046	0.901
6	Mean Z Value (SB)	GABA	-0.632	-1.865	0.602	-0.385	0.272
7	Mean Z Value (SB)	Glutamate/GABA	0.235	-0.041	0.510	0.570	0.085
8	Mean Z Value (ICA)	Age	0.014	0.001	0.026	0.658	**0.039**
9	Mean Z Value (ICA)	Glutamate	-0.047	-0.308	0.214	-0.145	0.689
10	Mean Z Value (ICA)	GABA	-0.421	-1.560	0.718	-0.289	0.419
11	Mean Z Value (ICA)	Glutamate/GABA	0.115	-0.169	0.398	0.313	0.378

*(β* = regression coefficient of the independent variable; CI = confidence interval for *β*; ICA = independent component analysis; *r* = Pearson’s correlation coefficient; SB = seed-based).

The concentrations of glutamate and GABA did not significantly correlate with age (*p* = 0.596 and *p* = 0.168, respectively), nor did they significantly correlate with the functional connectivity of the PCC/precuneus (*p* = 0.901 and *p* = 0.272, respectively).

**Fig 4:**
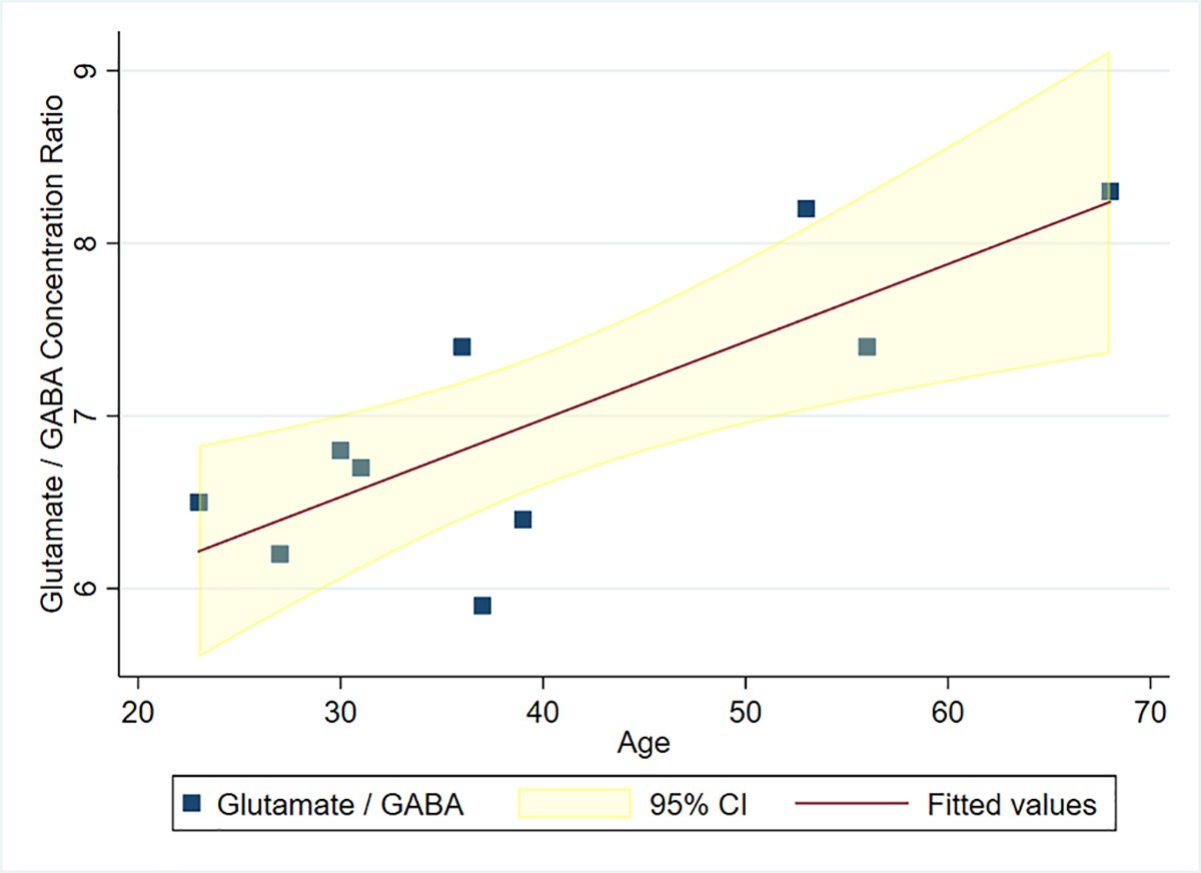
Glutamate/GABA concentration ratio in the PCC/precuneus vs age.

**Fig 5:**
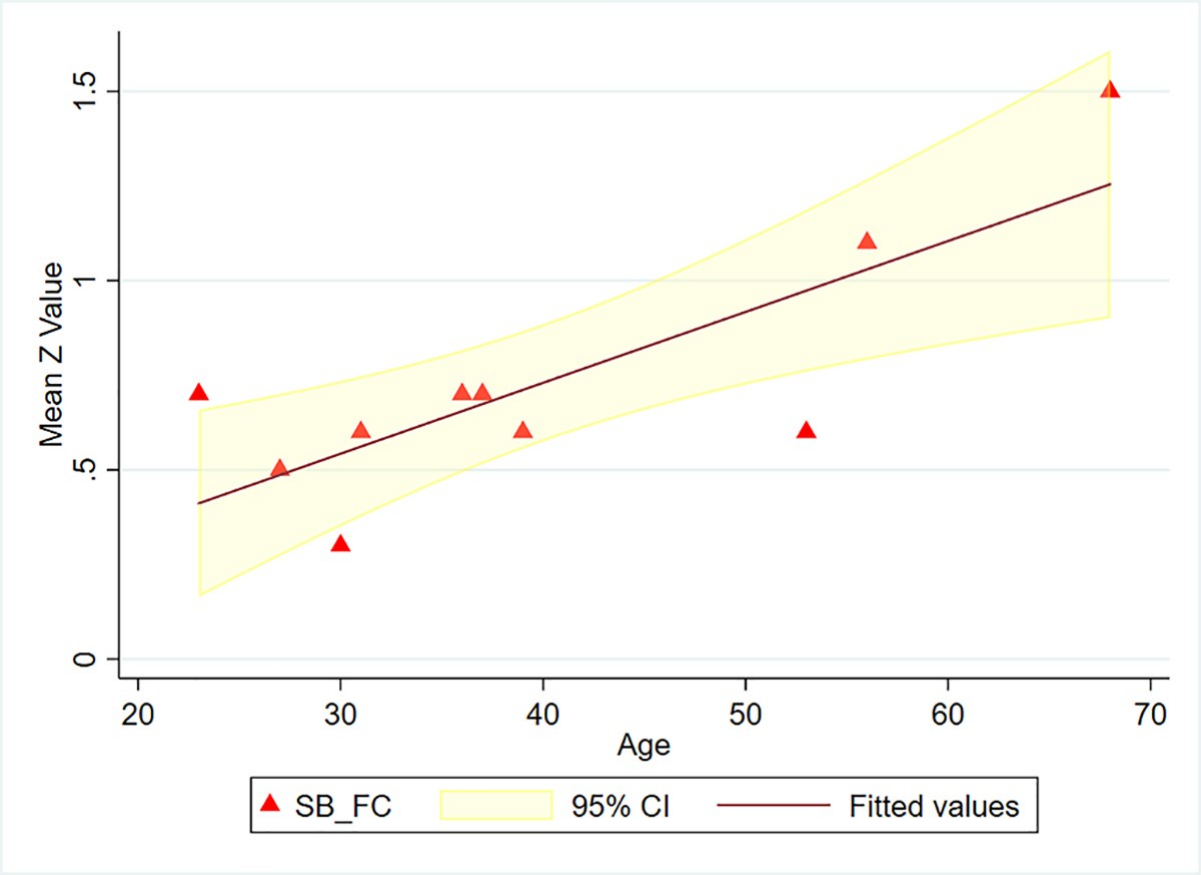
Mean functional connectivity of the PCC/precuneus vs age (seed-based). (Calculated as mean connectivity to the other ROIs expressed as Fisher transformed Z values; SB_FC = seed-based functional connectivity).

**Fig 6:**
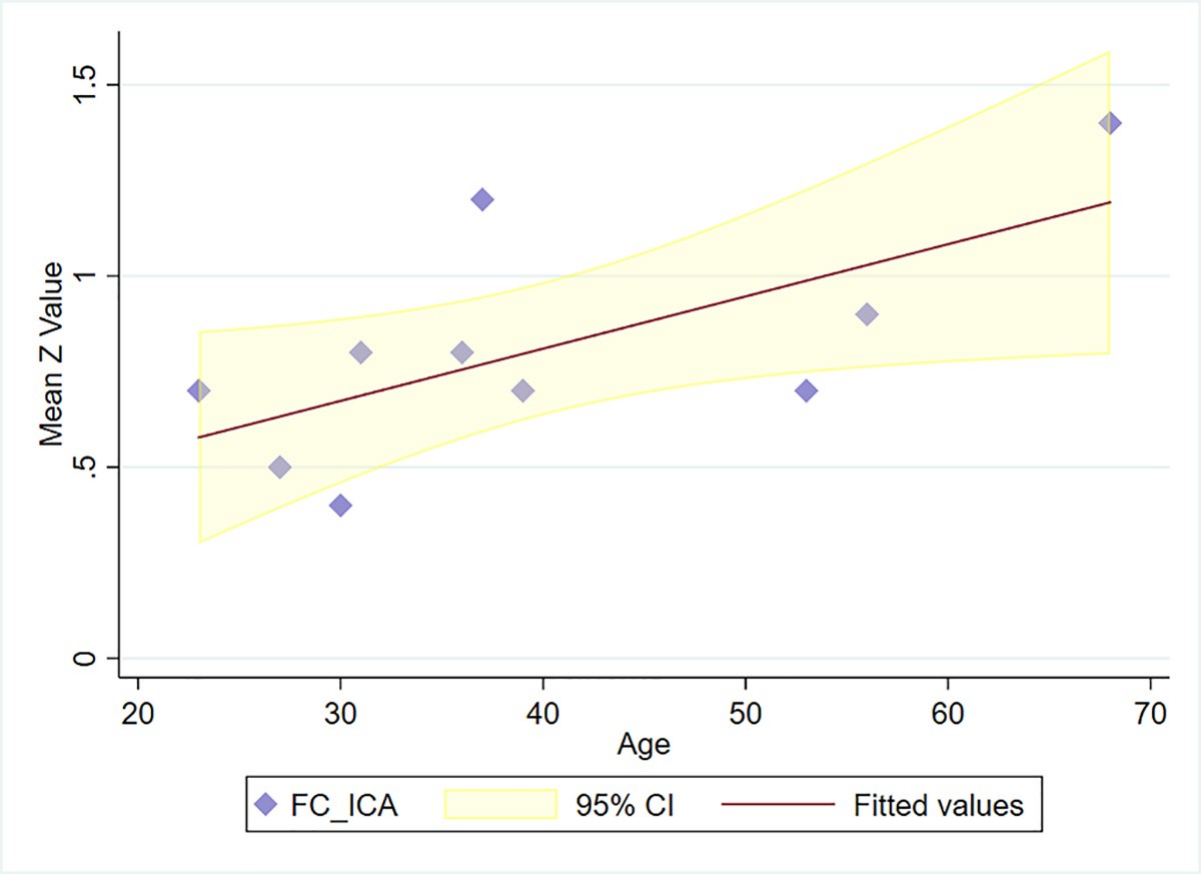
Mean functional connectivity of the PCC/precuneus vs age (ICA-based). (Calculated as mean connectivity to the other ROIs expressed as Fisher transformed Z values; ICA_FC = independent component analysis-based functional connectivity).

As shown in Fig 4, there was a positive correlation between the ratio of glutamate to GABA and age (*r* = 0.793, *p* = 0.006). A similar positive correlation (Fig 5) was found between the mean functional connectivity of the PCC/precuneus to the other 3 ROIs with age, as expressed in Z values (*r* = 0.802; *p* = 0.005). The concentration ratio glutamate/GABA did not significantly correlate with the functional connectivity of the PCC/precuneus (*p* = 0.085). In a similar manner to the seed-based results, the ICA-derived mean functional connectivity correlated significantly with age (*r* = 0.658; *p* = 0.039) (Fig 6).

## Discussion

In this study we evaluated the correlation of various measurable parameters of PCC/precuneus activity with age and with each other at 7T. Our results are in agreement with the 3T results of Arrubla et al, showing no significant correlation between the concentration of glutamate and DMN functional connectivity [14]. It is possible that a larger sample size would have produced a significant correlation between these metabolites and functional connectivity, as found in the study of Kapogiannis et al at 3T [13].

Kapogiannis et al also reported a positive correlation between the ratio of glutamate/GABA to functional connectivity of the DMN (as measured by the beta coefficients of the entire DMN following extraction by independent component analysis) and our results agree with this finding. It should be noted that no independent effect of age was found by Kapogiannis [13].

Recently, Gu et al explored the glutamate to GABA ratio in the PCC/precuneus in 65 healthy adults ranging in age between 17–49 years at 3T and also found no correlation to the intra-DMN functional connectivity, but found an inverse correlation to the functional connectivity between the DMN and the salience network [37]. The same group previously studied 24 healthy young subjects at 3T and found a positive correlation between GABA concentration in the PCC/precuneus and DMN deactivation, and a negative correlation with the concentration of glutamate. Interestingly, glutamate negatively correlated with age but GABA’s correlation with age was non-significant [38]. Few studies were conducted at 7T correlating fMRI with MRS glutamate and GABA. One such study, focusing on the occipital cortex was performed by Ip et al, who found no correlation between the BOLD signal and metabolite concentrations in the resting state [16].

We also demonstrated in our study that glutamate and GABA did not significantly correlate with age on their own, but their ratio did. Functional connectivity of the DMN also correlated with age. Suri et al quantified metabolites in an 8 cm^3^ voxel within the PCC/precuneus in 30 younger (20–40 years) and 151 cognitively healthy older adults (60–85 years) with 3T MRS [39]. GABA could not be reliably quantified. Glutamate was measured and was lower in older participants than in younger ones. Interestingly, the level of creatine was also higher in the older participants. This lends further support to our approach of quantifying metabolites relative to unsuppressed water (with partial volume correction) rather than relative to creatine.

The improved spectral resolution at 7T MRI enabled us to improve quantification of glutamate and GABA concentrations. Although no significant correlation with age was found for the concentrations of glutamate and GABA, when we examined the ratio of glutamate to GABA concentration, it significantly correlated with age, suggesting increased excitability in older individuals, which is speculated to underly the increased prevalence of certain neurological conditions with age, such as epilepsy [40]. This may be explained by previous studies demonstrating a decline in the number of GABAergic interneurons across several brain regions in humans and in animal models with healthy aging, in contrast to no such decline in excitatory neurons, thus increasing the ratio between excitatory to inhibitory neurons with age [40].

The most surprising finding in this study is the strong positive correlation between functional connectivity of the PCC/precuneus and age. It underscores the importance of taking age into account and including it as a covariate in future studies related to DMN functional connectivity. How can the findings of our study be explained? Many of the first studies of DMN functional connectivity were cross sectional and demonstrated reduced average connectivity with age when young and old age groups were compared [21]. More recent studies, some of which employed parcellation of DMN components, challenge this view.

Campbell et al studied the functional connectivity of various DMN components in 45 younger (18–29 years) and 39 older (60–83 years) healthy right-handed adults and found that while older adults had weaker functional connectivity in the ventral PCC, which is more strongly connected to the mesial temporal lobes, they had stronger connectivity in the dorsal PCC subsystem, which was mainly connected to the MPFC [41].

In another study by Huang et al involving 430 healthy elderly volunteers, the DMN was divided into anterior, ventral, and posterior subnetworks. Only the ventral subnetwork showed decline in functional connectivity with age [42]. As our study did not include temporal lobe ROIs, effectively interrogating the dorsal DMN subsystem, our results are in line with these recent studies.

Additionally, a study by Staffaroni et al assessed longitudinal functional connectivity in the DMN of 111 cognitively normal adults (ages 49–87, 46 women/65 men), and reported increasing connectivity within the DMN up to the age of 70 years, beyond which the connectivity declined [43]. Our results are based on a cohort younger than 70 years and are consistent with this finding. One possible theoretical explanation that has been suggested to explain this non-linear relationship with age described it as a compensatory process in response to neurodegeneration, until a tipping point is reached in which the increased connectivity cannot be sustained any longer [43].

This study is limited by its small sample size and should be regarded exploratory in nature. Nonetheless, our findings are consistent with several previous studies of subjects of comparable age range. In addition, we calculated functional connectivity both using a seed-based approach with localization based on prior studies, which has the advantage of the possibility of future comparison to various patient groups, and using a data-driven ICA approach, thus conferring greater validity to our results.

## Conclusions

The ratio of glutamate/GABA in the PCC/precuneus and its functional connectivity with the other main DMN nodes, as measured in this 7T study, independently correlate with age in healthy adults but not with each other. Further multimodal evaluation of these metabolites with DMN functional connectivity in conditions known to affect this brain region (e.g. Alzheimer’s disease, epilepsy) at 7T would be worthwhile exploring.

## Supporting information

S1 Fig Overlapping MRS voxel localizations of the 10 participants. (Normalized to standard space using FSL-FLIRT with 12 degrees of freedom; left–sagittal; right—axial).
(TIF)

S2 Fig Clusters of the ICA2 component passing the p-FDR<0.05 threshold. (The colormap corresponds to T values).
(TIF)

S3 Fig Clusters of the ICA5 component passing the p-FDR<0.05 threshold. (The colormap corresponds to T values).
(TIF)

S1 Table Clusters extracted from ICA analysis. (x, y, z values represent the MNI coordinates of peak activation).
(DOCX)

S2 Table Quantification of all metabolites acquired consistently with CRLB < 20%. (CRLB = Cramér–Rao lower bounds; CSF = cerebrospinal fluid; GABA = γ-amino butyric acid GLUT = glutamate; GLN = glutamine; GM = gray matter; GSH = glutathione; INS = inositol; NAA = N-acetylaspartic acid; TCr = total creatine (creatine + phosphocreatine); WM = white matter).
(DOCX)

## Abbreviations

CRLB: Cramér–Rao lower bounds
DMN: default mode network
GABA: γ-amino butyric acid
GSH: glutathione
ICA: independent component analysis
IPL: inferior parietal lobule
MP2RAGE: magnetization prepared 2 rapid gradient echoes
MPFC: mesial prefrontal cortex
PCC: posterior cingulate cortex
STEAM: stimulated echo acquisition mode
