# Supplementary figures and images for: Resting-state functional connectivity and quantitation of glutamate and GABA of the PCC/precuneus by magnetic resonance spectroscopy at 7T in healthy individuals
Source: PLoS One. 2020 Dec 29;15(12):e0244491. doi: 10.1371/journal.pone.0244491 (PMC7771854; doi:10.1371/journal.pone.0244491)

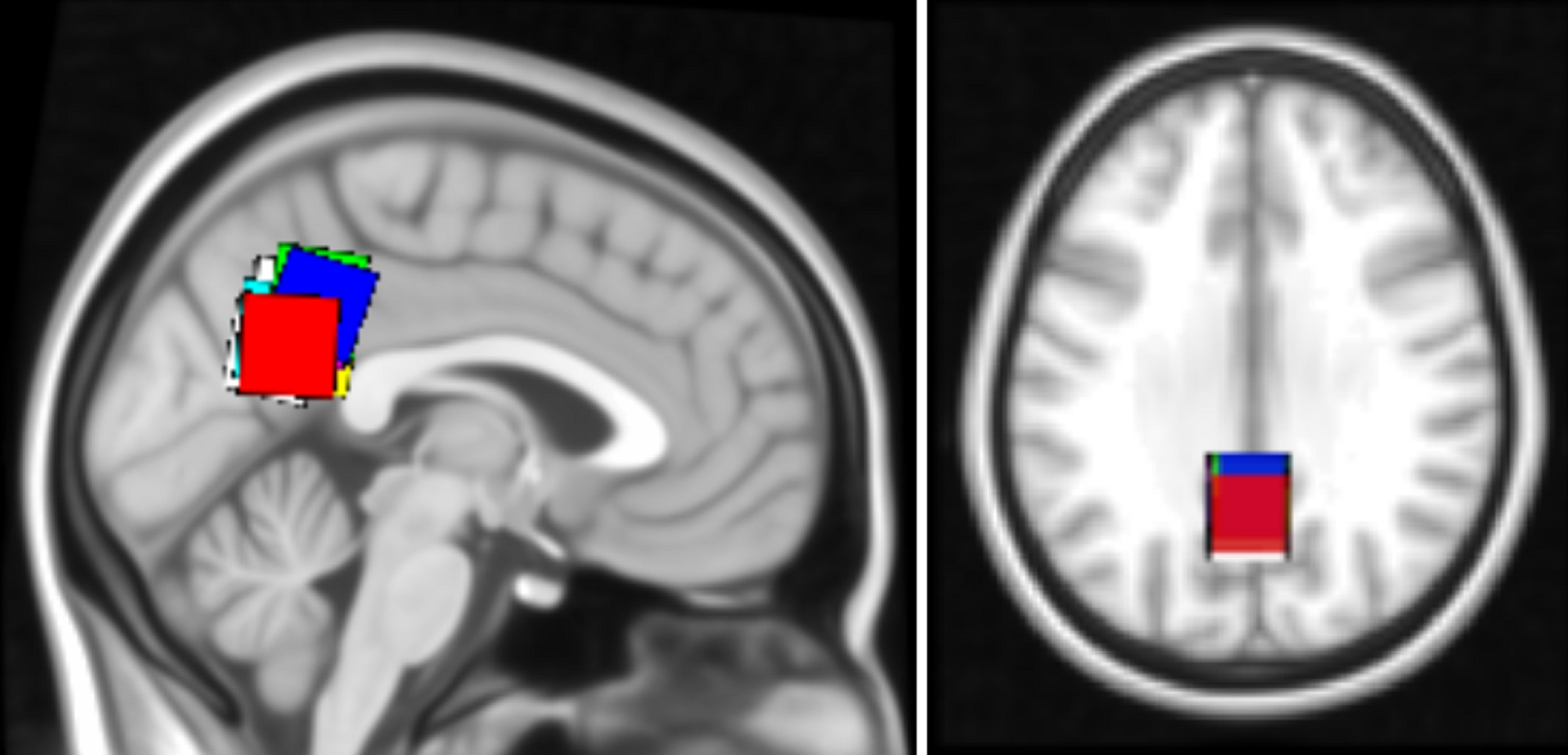

Supplement: S1 Fig — (Normalized to standard space using FSL-FLIRT with 12 degrees of freedom; left–sagittal; right—axial). (TIF) [file pone.0244491.s001.tif]

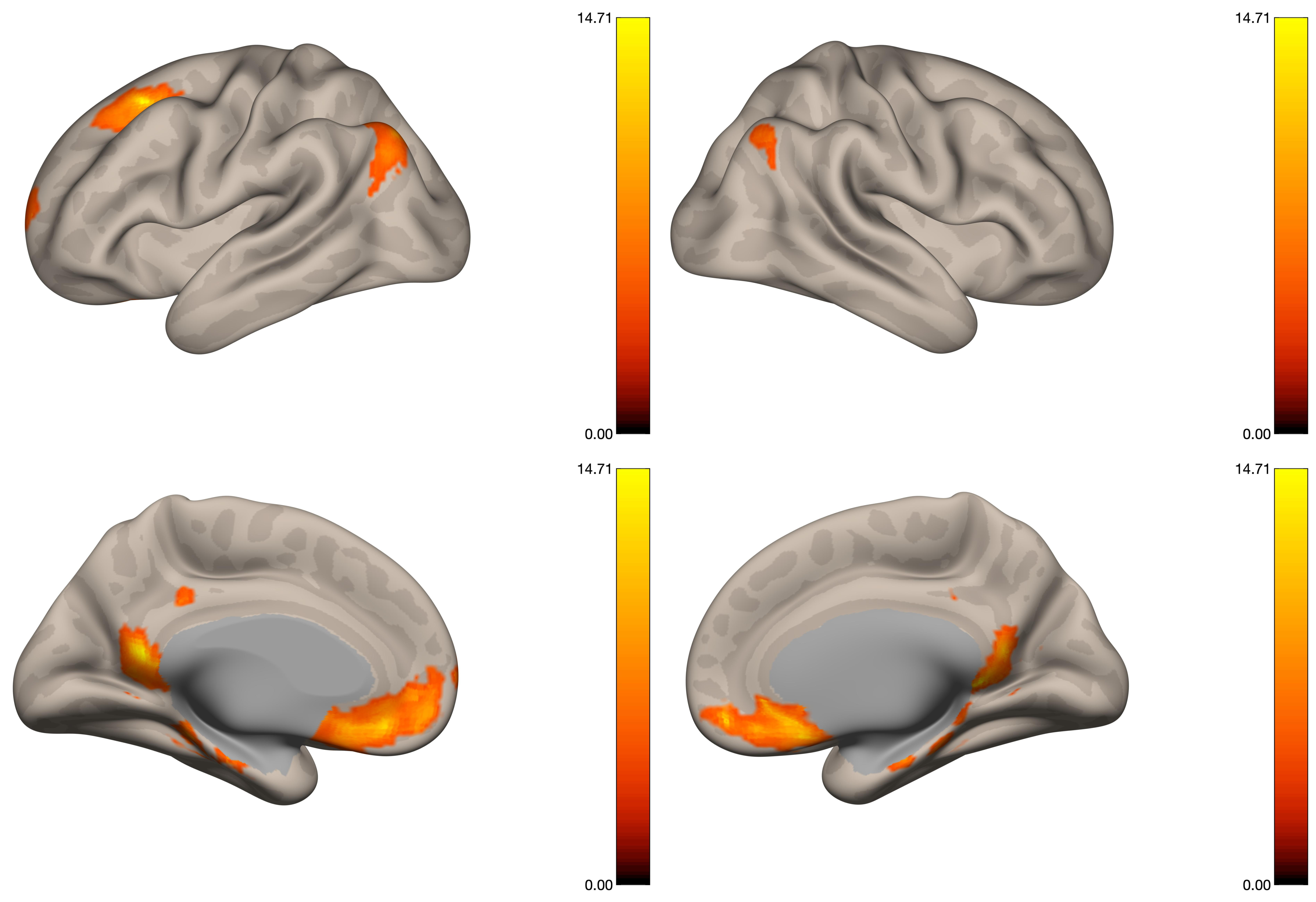

Supplement: S2 Fig — (The colormap corresponds to T values). (TIF) [file pone.0244491.s002.tif]

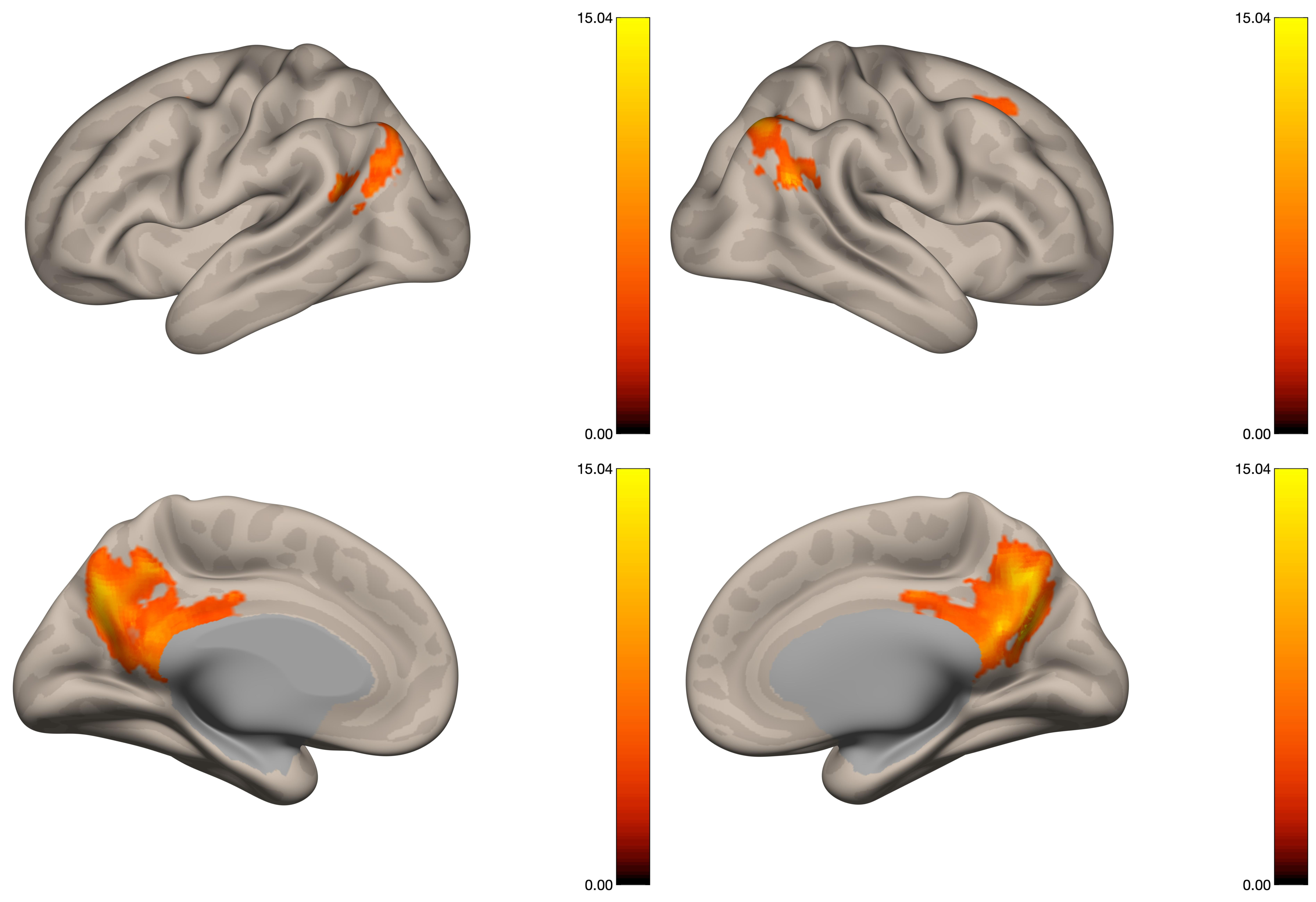

Supplement: S3 Fig — (The colormap corresponds to T values). (TIF) [file pone.0244491.s003.tif]
