# Supplementary material for: Resting-state functional connectivity and quantitation of glutamate and GABA of the PCC/precuneus by magnetic resonance spectroscopy at 7T in healthy individuals
Source: PLoS One. 2020 Dec 29;15(12):e0244491. doi: 10.1371/journal.pone.0244491 (PMC7771854; doi:10.1371/journal.pone.0244491)
Supplement: S1 Table — (x, y, z values represent the MNI coordinates of peak activation). (DOCX) [file pone.0244491.s004.docx]

**Table 1S – Clusters extracted from ICA analysis**

(x, y, z values represent the MNI coordinates of peak activation)
