# Supplementary material for: Resting-state functional connectivity and quantitation of glutamate and GABA of the PCC/precuneus by magnetic resonance spectroscopy at 7T in healthy individuals
Source: PLoS One. 2020 Dec 29;15(12):e0244491. doi: 10.1371/journal.pone.0244491 (PMC7771854; doi:10.1371/journal.pone.0244491)
Supplement: S2 Table — (CRLB = Cramér–Rao lower bounds; CSF = cerebrospinal fluid; GABA = γ-amino butyric acid GLUT = glutamate; GLN = glutamine; GM = gray matter; GSH = glutathione; INS = inositol; NAA = N-acetylaspartic acid; TCr = total creatine (creatine + phosphocreatine); WM = white matter). (DOCX) [file pone.0244491.s005.docx]

**Table 2S – Quantification of all metabolites acquired consistently with CRLB < 20%
(CRLB = Cramér–Rao lower bounds; CSF = cerebrospinal fluid; GABA = γ-amino butyric acid GLUT = glutamate; GLN = glutamine; GM = gray matter; GSH = glutathione; INS = inositol; NAA = N-acetylaspartic acid; TCr = total creatine (creatine + phosphocreatine); WM = white matter)**
